# Programmed death of intestinal epithelial cells in neonatal necrotizing enterocolitis: a mini-review

**DOI:** 10.3389/fped.2023.1199878

**Published:** 2023-06-05

**Authors:** Shuo Yang, Xin Wei, Yuting Zhong, Conglu Guo, Xinzhu Liu, Zhibin Wang, Ye Tu

**Affiliations:** ^1^ Department of Pharmacy, Shanghai East Hospital, School of Medicine, Tongji University, Shanghai, China; ^2^ Department of Critical Care Medicine, School of Anesthesiology, Naval Medical University, Shanghai, China; ^3^ Department of Clinical Pharmacy, Xinhua Hospital, Shanghai Jiaotong University School of Medicine, Shanghai, China

**Keywords:** necrotizing enterocolitis, intestinal epithelial barrier, intestinal epithelial cells, programmed cell death, treatment strategies

## Abstract

Necrotizing enterocolitis (NEC) is one of the most fatal diseases in premature infants. Damage to the intestinal epithelial barrier (IEB) is an important event in the development of intestinal inflammation and the evolution of NEC. The intestinal epithelial monolayer formed by the tight arrangement of intestinal epithelial cells (IECs) constitutes the functional IEB between the organism and the extra-intestinal environment. Programmed death and regenerative repair of IECs are important physiological processes to maintain the integrity of IEB function in response to microbial invasion. However, excessive programmed death of IECs leads to increased intestinal permeability and IEB dysfunction. Therefore, one of the most fundamental questions in the field of NEC research is to reveal the pathological death process of IECs, which is essential to clarify the pathogenesis of NEC. This review focuses on the currently known death modes of IECs in NEC mainly including apoptosis, necroptosis, pyroptosis, ferroptosis, and abnormal autophagy. Furthermore, we elaborate on the prospect of targeting IECs death as a treatment for NEC based on exciting animal and clinical studies.

## 1. Introduction

NEC is the most common and fatal gastrointestinal emergency in the neonatal intensive care unit (NICU) (1, 2), and 90% of these infants who develop NEC are preterm (3, 4). Epidemiological results show that the total incidence of NEC in NICU infants is 2% to 5%, among which the morbidity and mortality very low birth weight (VLBW; <1,500 g) neonates are 4.5%−8.7% and 20%−30%, respectively, while the mortality rate in extremely low birth weight (ELBW; <1,000 g) infants is even as high as 30% to 50.9% (5–7). A better understanding of the pathogenesis of NEC is the key to finding better diagnostic, preventive, and therapeutic strategies.

The intestinal tract of premature infants is characterized by incomplete development of immune defense function, and impairment of the intestinal epithelial barrier (IEB) function exacerbated after experiencing hypoxia and hypothermia (8, 9). The disruption of IEB is a key factor in the development of intestinal inflammation (10), while increased intestinal permeability caused by IEB injury can be further amplified by intestinal inflammation, thus forming a positive feedback loop (10). Furthermore, the aggravated intestinal permeability and altered intestinal microbiome work together to cause the transfer of bacteria or bacterial products across the IEB, triggering a more intense inflammatory response, and even adverse consequences such as NEC, late-onset sepsis (LOS), intestinal perforation, and death (11–13). Therefore, it has been proposed that NEC evolves from the destruction of the IEB (14) and further develops into LOS in the imbalance between IEC damage and repair (15).

The anti-permeability of IEB is mainly maintained and regulated by intestinal epithelial cells (IECs) and the tight junction (TJ) formed between IECs in the apical region (16). The intestinal epithelium is a single-cell layer structure composed of multiple cells differentiated from intestinal stem cells (ISCs) (17). Eighty percent of the IECs are absorptive villi epithelial cells, which are responsible for maintaining the barrier function of the IEB, while the Paneth cells located in the basement of the intestinal crypts are considered to be “reserve ISCs” and play an important role in the repair of the intestinal epithelium (18). Severe injury of IEB and increased intestinal permeability are the most prevalent microscopic phenotypes in NEC (19). More importantly, increased intestinal permeability occurs before the appearance of NEC signs (20), and the destruction of IEB in turn makes premature infants susceptible to NEC (21). Therefore, understanding the cellular damage mechanism of IECs is key to revealing IEB dysfunction.

## 2. Death of IECs

**Figure 1:**
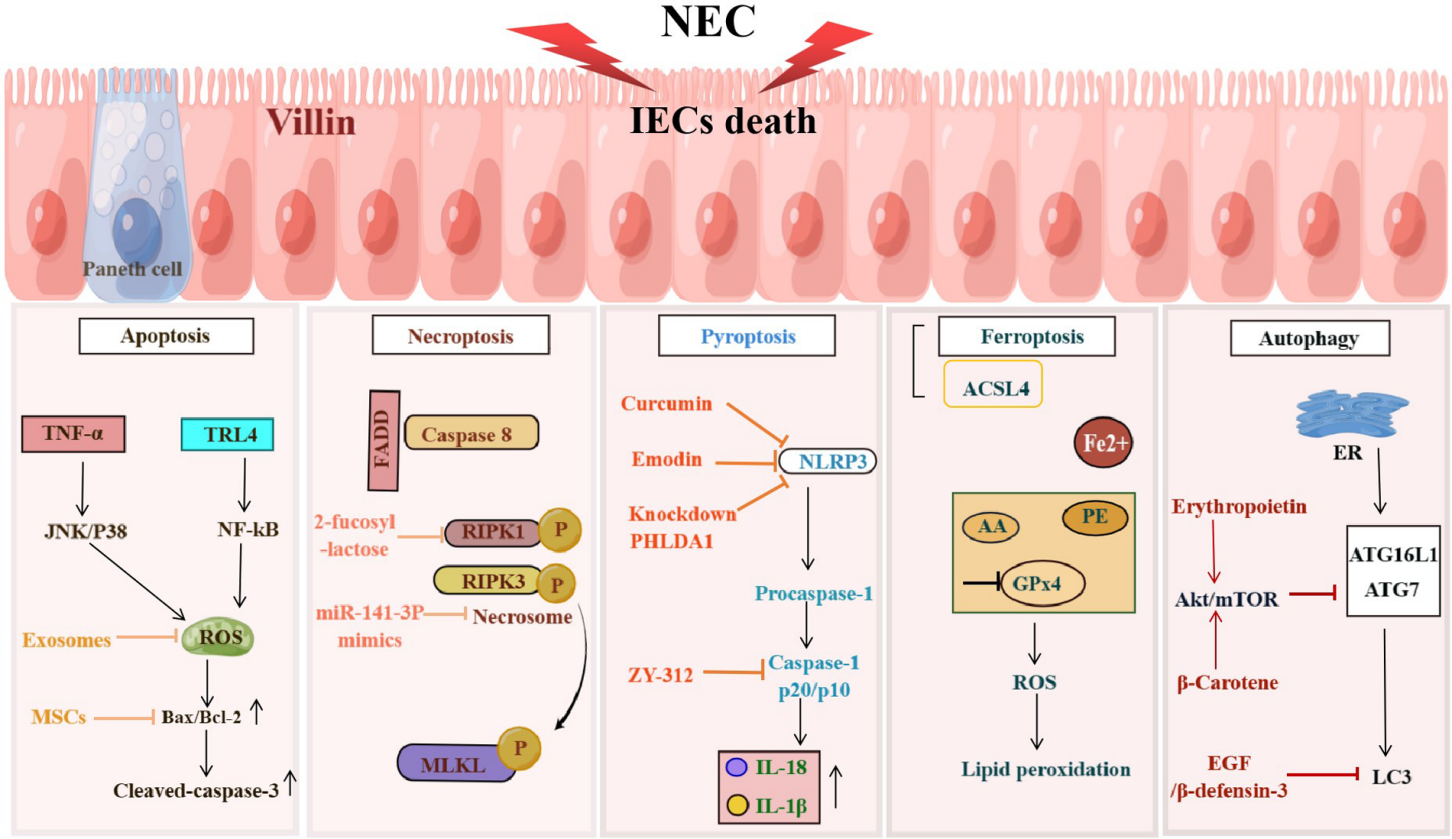
NEC induces programmed death of IECs. Programmed cell death including apoptosis, necroptosis, pyroptosis, ferroptosis and autophagy occurs in IECs during the development of NEC, resulting in the damage to the IEB. The figure shows the signaling pathways in different death types of IECs.

The definition of NEC is derived from the fact that IECs die in this disease (22). Therefore, one of the most basic problems in the field of NEC research is how do IECs die. Answering this question is essential for elucidating the pathogenesis of NEC. The currently known death modes of IECs in NEC mainly include apoptosis, necroptosis, pyroptosis, ferroptosis, and abnormal autophagy (Figure 1).

### 2.1. IECs apoptosis

Intestinal epithelial cells have an extremely high rate of self-renewal, and their self-clearance at the end of the life cycle in an apoptotic manner is essential. However, excessive apoptosis may exacerbate the disruption of the intestinal barrier, allowing more microorganisms to enter the submucosa and producing large amounts of cytokines, and further inducing IECs apoptosis (23). For example, TNF-α leads to apoptosis of IECs by inducing mitochondrial reactive oxygen species production and activating the JNK/p38 signaling pathway (24). In the neonatal rat NEC model, the mRNA ratio of Bax/Bcl-2 and the protein expression level of Bax were significantly increased in the damaged ileum tissue. Besides, the number of cleaved-caspase-3 positive cells was also markedly increased in the epithelial cells of the terminal ileum. Considering that the ileum is the most vulnerable intestinal tissue in NEC, these results indicate the existence of abnormal apoptosis of IECs in NEC (25).

In the small intestinal tissue of neonatal mice, TLR4 activates the NF-*κ*B signaling pathway and leads to increased apoptosis of intestinal cells (26). In contrast, LPS-induced IECs apoptosis and the incidence of NEC were markedly reduced in TLR4 knockout mice (27). In addition, TLR4-induced endoplasmic reticulum stress also led to increased apoptosis of ISCs (28), thereby affecting the repair of the intestinal epithelium. An increased mRNA ratio of Bax/Bcl-2 was also observed in the resected ileal tissues from NEC infants (29). A large number of TUNEL-positive cells were observed in the apical villus cells, and these cells also showed positive nitrotyrosine staining, suggesting that the apoptosis of IECs may also be associated with the formation of peroxynitrite caused by NO (30).

Despite species differences, human breast milk-derived exosomes have been shown to prevent H_2_O_2_-induced cytotoxicity and reduce oxidative stress damage in IECs in a mouse NEC model (31). Oxidative stress is one of the main factors that increase the expression of the tumor suppressor p53, which promotes apoptosis (32). Breast milk-derived exosomes were further shown to regulate IECs injury by targeting p53 through the delivery of miRNA-125b (33).

In addition, stem cell therapy has been gradually applied in NEC treatment in recent years. Stem cells have self-renewal ability, multi-directional differentiation, and good applicability. Injecting stem cells into NEC model rats can effectively inhibit apoptosis, reduce inflammation and protect the intestinal barrier (34, 35). Mesenchymal stem cells (MSCs) have been shown to reduce the incidence and severity of experimental NEC in rats (36, 37). Intraperitoneal injection of amniotic fluid-derived mesenchymal stem cells (AF-MSCs) can suppress Bax expression by activating Bib and upregulating CHOP, thereby resisting the apoptosis of IECs caused by endoplasmic reticulum stress (38, 39). Stem cell therapy has been successfully applied to clinical cases in 2019 (40), but more evidence is still needed to further elucidate the therapeutic mechanisms of stem cells in NEC to achieve breakthroughs in clinical trials.

### 2.2. IECs necroptosis

Although apoptosis has long been recognized as the major mode of IECs death in NEC (25, 41). However, intestinal inflammation is an important hallmark of NEC (21), whereas apoptosis, as a non-inflammatory type of cell death (42), seems insufficient to explain the rapid and intense inflammatory response in NEC, suggesting that apoptosis is more like a consequence than a cause of NEC (22). Programmed necrosis is another highly regulated type of programmed cell death also known as necroptosis, which is a highly inflammatory type of cell death independent of caspase activation (43). Necroptosis is characterized by the formation of necrosome upon activation of receptor-interacting protein kinases (RIPK1 and RIPK3), which in turn activate mixed-lineage kinase-like (MLKL). Phosphorylated MLKL oligomerizes and translocates to the cell membrane, resulting in cell membrane rupture and release of damage-associated molecular patterns (DAMPs) to trigger an inflammatory cascade response (43). Necroptosis is associated with the pathogenesis of a variety of mucosal inflammatory diseases (44), including inflammatory bowel disease and allergic colitis in children, and lethal ileitis during intestinal development (45, 46). Recent studies have reported that necroptosis is observed in IECs of human and mouse NEC models, especially in differentiated villous epithelial cells. Blocking necroptosis by inhibitors or gene knockdown can reduce the pathological damage of IEB in NEC mice (22), suggesting that necroptosis of IECs plays an important role in NEC-related IEB injury. Interestingly, in addition to mediating apoptosis of IECs, TLR4 also mediates necroptosis in the development of NEC (47).

The cell types of IECs affected by apoptosis and necroptosis are different in the pathogenesis of NEC. Apoptosis occurs mainly in the intestinal crypt epithelial cells (e.g., Paneth cells) (28, 33), while necroptosis mainly occurs in intestinal epithelial villi cells (22). Differences in the death types of IECs may be related to their different biological roles in the pathogenesis of NEC. Apoptosis of ISCs can lead to intestinal mucosal epithelial repair disorder, affecting the recovery of IEB function (29). Necroptosis explains the rupture of intestinal villi in NEC, which facilitates microbial translocation across the IEB and promotes the development of LOS (22). In addition, necroptosis that occurs in infiltrating lymphocytes in the neonatal intestine can further promote the loss of IECs (22). Therefore, necroptosis of IECs may be a more direct pathological death mode in IEB lesions due to NEC.

2-fucosyl lactose isolated from breast milk can effectively prevent the activation of necroptosis-characteristic genes in the ileum tissue of premature infants with NEC (22). This result partially explains why infants fed breast milk have a lower risk of NEC than infants fed formula milk, which is due to the lack of 2-fucose lactose in formula milk. miR-141-3p mimics can inhibit the up-regulation of necroptosis-related molecules and the interaction of RIPK1 and RIPK3 in LPS-induced Caco-2 cells (48), suggesting that miR-141-3p protects IECs from cellular injury by inhibiting RIPK1-mediated necroptosis and inflammatory cytokine release, providing a perspective for exploring the pathogenesis of NEC.

### 2.3. IECs pyroptosis

Pyroptosis is another inflammatory mode of programmed cell death reported in recent years (49). It mainly relies on inflammasomes to activate caspase-1, resulting in the shearing of gasdermin D (GSDMD). Subsequently, the cleaved GSDMA translocates to the cell membrane and forms pores through polymerization, leading to cell membrane rupture and cell death, accompanied by the release of large amounts of cytokines (50). In addition, activated caspase-1 also cleaves the precursors of IL-1β and IL-18 to form mature IL-1β and IL-18, which are then released into the extracellular and aggravate the inflammatory response (51). *Cronobacter sakazakii* has been reported to induce NEC. The underlying mechanism is to promote the activation of NLRP3 inflammasome by activating TLR4/MyD88/NF-kB signaling pathway, resulting in the up-regulation of downstream caspase-1 expression and the increase of IL-1β release, and ultimately inducing pyroptosis of intestinal cells (52). Significantly increased mRNA expression of IL-1β, IL-18 and NLRP3, and protein expression of caspase-1 p20/p10 were also observed in the ileocecal region of NEC model mice (53). Therefore, there seems to be the possibility of intestinal epithelium pyroptosis in the animal models of NEC, but more detailed pathological diagnosis and anti-pyroptosis treatment studies still need to be further carried out. In addition, increased levels of IL-1β were detected in the peripheral blood of infants with NEC, and the expression of IL-1β and caspase-1 was also observed in the intestinal epithelium (54).

Curcumin attenuates NLRP3/caspase-1-induced pyroptosis by activating the SIRT1/NRF2 signaling pathway or inhibiting TLR4 expression, thereby reducing intestinal injury in the rat model of NEC (55). Emodin has also been reported to reduce intestinal pyroptosis in NEC neonatal rats by inhibiting the NLRP3/IL-1β pathway (56). Recently, PHLDA1 was found to be highly expressed in the intestinal tissues of NEC mice. In sh-PHLDA1 transfected mice with NEC, the survival rate was increased, and intestinal inflammation, oxidative stress, and cell scorching were also improved significantly (57). Knockdown of PHLDA1 was shown to alleviate the NEC phenotype by activating Nrf2 to inhibit NLRP3 activation and pyroptosis (57). Fan et al. isolated a novel probiotic candidate strain from fecal samples of healthy infants and characterized it as *Bacteroides fragilis* strain ZY-312 (58). ZY-312 can reduce *Cronobacter sakazakii*-induced pyroptosis by inhibiting caspase-1 and decreasing IL-1β in NEC rats. Interestingly, ZY-312 also reversed the increased levels of caspase-3 and Bax/Bcl-2, suggesting that ZY-312 could simultaneously block apoptosis induced by *Cronobacter sakazakii* (58). As a promising probiotic agent, ZY-312 has great utility for the prevention and treatment of NEC by reducing dual cell death forms (pyroptosis and apoptosis) to restore IEB function and alleviate local inflammatory response.

### 2.4. IECs ferroptosis

Ferroptosis is an iron-dependent programmed cell necrosis characterized by lipid peroxidation and membrane damage (59). In recent years, several studies have demonstrated the presence of ferroptosis in IEB pathological injury in a variety of colorectal diseases including ulcerative colitis (60, 61), Crohn's disease (62), and intestinal ischemia/reperfusion injury (63, 64). Ferroptosis leads to elevated levels of lipid peroxidation (LPO)by inhibiting the activation of glutathione peroxidase 4 (GPx4) and the oxidation of arachidonic acid (AA) and its esterified phosphatidylethanolamine (PE) (63, 65), resulting in the death of IECs and disruption of the intestinal mechanical barrier (66). Antioxidant enzyme activity is lower in neonates compared to adults, making infants more susceptible to oxidative stress (67). Peroxidation-induced ROS accumulation is thought to produce a strong pro-inflammatory effect in NEC-associated ferroptosis (68). Bioinformatic analysis and wet experiments have demonstrated the involvement of ferroptosis in NEC and found that ACSL4, a key regulator of ferroptosis execution, may participate in NEC by activating NEC-related Toll-like receptor signaling pathway to induce IECs death and immune cell activation (69). Meanwhile, ACSL4 is also involved in autophagy, pyroptosis, apoptosis, hypoxia, and inflammation of NEC (69). However, there is still a lack of anti-ferroptosis therapy in NEC. There is currently little evidence to support the ferroptosis of IECs in NEC, and the specific mechanism needs to be further confirmed, which will also provide new insights into the treatment of NEC.

### 2.5. IECs autophagy

Autophagy is a process of lysosome-dependent degradation of proteins and organelles and interacts with apoptosis to synergistically eliminate aging, redundant or damaged cells (70). Excessive autophagy has been identified as a risk factor for NEC development (71). Hackam et al. previously reported that gene expression levels of *TLR4*, *ATG7*, *LC3*, *ATG16* and *Beclin1* were significantly increased in the fetal intestine compared to full-term infants, and that intestinal tissues obtained from aborted fetuses also showed significantly increased staining for LC3 within the intestinal epithelium (72). Human *β*-defensin 3 (hBD3) treatment inhibits excessive autophagy in IECs may through the CXCR4 signaling pathway. Meanwhile, hBD3 significantly reduces the expression of autophagy-related proteins (Beclin1, LC3 and p62) and inflammatory cytokines, leading to improved migration of IECs and intestinal mucosal integrity, as well as a reduction in mortality in NEC rats (73). Similarly, erythropoietin in breast milk protects IECs from excessive autophagy and apoptosis in NEC mice via the Akt/mTOR and MAPK/ERK signaling pathway (74). Epidermal growth factor (EGF) treatment attenuates NEC injury by modulating intestinal autophagy in rat models (71, 75). *β*-carotene also attenuates LPS-induced apoptosis and autophagy in IECs by activating the PI3K/AKT/mTOR signaling pathway (76). All these studies suggest that IECs autophagy and apoptosis play an important role in NEC pathological damage, and modulating IECs autophagy can be used as an effective complement to anti-apoptotic therapy for NEC.

## 3. Conclusion and outlook

How does the pathological IECs death mechanism interact and induce increased intestinal permeability, leading to intestinal barrier dysfunction? Can targeting one of these cell death mechanisms as a therapeutic strategy provide effective treatment for infants with NEC? Although these problems remain questionable, understanding the mechanisms of IECs' death has brought new insights to explore new treatments (77). In this review, we highlight the important role of IECs death mechanisms in the process of NEC and review potential therapeutic approaches to alleviate NEC by preventing IECs death in recent years (Table 1). Future breakthroughs in NEC treatment should focus on finding drugs or interventions that can effectively protect IEB. Mechanistically, it is desirable that these drugs directly target the programmed death of IECs and the associated severe IEB damage caused by inflammation.

**Table 1:** Therapies and drugs targeting different IECs death pathway.

Cell Death	Model	Treatments	Key molecular mechanism
Apoptosis	*In vitro* IEC-6 cells	Human breast milk-derived exosomes	Target and inactivate the apoptosis-inducer p53 through delivering microRNA-125b;
			Reduce oxidative stress-related injury (33)
	*In vivo* mice NEC model	Mesenchymal stem cells (MSCs)	AF-MSCs antagonize the ER stress by activating Bip and upregulating CHOP, inhibiting the expression of Bax, and stimulating the expression of Bcl-2 (38)
Necroptosis	*In vivo* mice NEC model	Human breast milk, or 2 fucosyl lactose	Inhibit downstream of RIPK1;
			Inhibit synergetic necroptosis–apoptosis cross-talk (22)
	*In vitro* LPS-treated Caco-2 cells	MiR-141-3p	Suppress RIPK1-mediated inflammation and necroptosis (48)
Pyroptosis	*In vivo* the rat NEC model	Curcumin	Activate SIRT1/NRF2;
			Inhibit the TLR4 signaling pathway (55)
	*In vivo* the rat NEC model	Emodin	Inhibit NLRP3-IL-1β signaling pathway (56)
	*In vivo* neonatal mouse model of NEC	Knockdown of PHLDA1	Activate Nrf2 signaling pathway;
			Inhibit NLRP3 activation (57)
	*In vivo* neonatal rat model*;*	Bacteroides fragilis strain ZY-312	Inhibit caspase-1 and reduce IL-1β levels;
	*In vitro* Caco-2 cell lines		
			Decrease expression of caspase-3, Bax and increase expression of Bcl-2 (Reverse the apoptosis) (58)
Ferroptosis	Human intestinal tissue samples;	Absence	ACSL4 may induce ferroptosis and immune cell activation through NEC related signaling pathway (69)
	*In vivo* mice NEC model;		
	*In vitro* Caco-2 cell lines		
Autophagy	*In vitro* IEC-6 and Caco2 enterocytes;	Human β-defensin-3	Inhibit migration;
			CXCR4 signaling pathway (did not provide direct evidence) (73)
	*In vivo* rat model of NEC		
	*In vitro* IEC-6 cells;	Erythropoietin (Epo) from breast milk	Reduce apoptosis through the MAPK/ERK pathway;
	*In vivo* rat NEC experimental model		
			Decreased autophagy via the Akt/mTOR signaling pathway (74)
	*In vitro* IEC-6 cells;	Epidermal growth factor (EGF)	Reduce the expression of autophagy-related proteins (Beclin1, LC3 and p62) (75)
	*In vivo* the rat NEC model		
	*In vitro* IEC-6 cells	β-Carotene	Attenuate apoptosis and autophagy via PI3K/AKT/mTOR signaling pathway (76)
